# The Expanding Family of Bone Marrow Homing Factors for Hematopoietic Stem Cells: Stromal Derived Factor 1 Is Not the Only Player in the Game

**DOI:** 10.1100/2012/758512

**Published:** 2012-06-04

**Authors:** Mariusz Z. Ratajczak, ChiHwa Kim, Anna Janowska-Wieczorek, Janina Ratajczak

**Affiliations:** ^1^Stem Cell Biology Program at the James Graham Brown Cancer Center, University of Louisville, Louisville, KY 40202, USA; ^2^Department of Physiology, Pomeranian Medical University, 70-111 Szczecin, Poland; ^3^Department of Medicine, University of Alberta, Edmonton, AB, Canada T6G 2G3

## Abstract

The **α**-chemokine stromal derived factor 1 (SDF-1), which binds to the CXCR4 and CXCR7 receptors, directs migration and homing of CXCR4^+^ hematopoietic stem/progenitor cells (HSPCs) to bone marrow (BM) and plays a crucial role in retention of these cells in stem cell niches. However, this unique role of SDF-1 has been recently challenged by several observations supporting SDF-1-CXCR4-independent BM homing. Specifically, it has been demonstrated that HSPCs respond robustly to some bioactive lipids, such as sphingosine-1-phosphate (S1P) and ceramide-1-phosphate (C1P), and migrate in response to gradients of certain extracellular nucleotides, including uridine triphosphate (UTP) and adenosine triphosphate (ATP). Moreover, the responsiveness of HSPCs to an SDF-1 gradient is enhanced by some elements of innate immunity (e.g., C3 complement cascade cleavage fragments and antimicrobial cationic peptides, such as cathelicidin/LL-37 or **β**2-defensin) as well as prostaglandin E2 (PGE2). Since all these factors are upregulated in BM after myeloblative conditioning for transplantation, a more complex picture of homing emerges that involves several factors supporting, and in some situations even replacing, the SDF-1-CXCR4 axis.

## 1. Introduction

The *α*-chemokine stromal derived factor 1 (SDF-1), which binds to the seven-transmembrane-spanning G_*α*I_ protein-coupled receptor CXCR4 is, as has been demonstrated by several investigators, unique among the family of chemokines, because it directly chemottracts hematopoietic stem/progenitor cells (HSPCs) [1–3]. Since CXCR4 is expressed on both long- and short-term repopulating hematopoietic stem cells (HSCs), as well as hematopoietic progenitors, SDF-1 plays an important role in regulating trafficking of these cells and their homing after transplantation to BM, and is later involved in their active retention in BM stem cell niches [2–5].

However, while a role for the SDF-1-CXCR4 axis in retention of HSPCs in BM under steady-state conditions is undisputed, its exclusive role in stem cell homing has been challenged by several observations that support the existence of SDF-1-CXCR4-independent homing [6–8]. In particular, it has been reported that; (i) CXCR4^−/−^ fetal liver HSPCs home to BM in an SDF-1-independent manner [6], (ii) homing of murine HSPCs made refractory to SDF-1 by incubation and coinjection with a CXCR4 receptor antagonist (AMD3100) is normal or only mildly reduced [7], (iii) HSPCs in which CXCR4 has been knocked down by means of an SDF-1 intrakine strategy are able to engraft [8], and finally (iv) myeloablative conditioning for transplantation induces a highly proteolytic microenvironment in BM that leads to proteolytic degradation of SDF-1, and thus may severely attenuate its chemotactic gradient [9].

All this evidence strongly suggests the involvement of other factors that support homing of HSPCs. In this paper, we will present cumulative evidence that gradients of the bioactive sphingophospholipids, such as sphingosine-1-phosphate (S1P) [10–12] and ceramide-1-phosphate (C1P) [9, 13, 14], which are products of membrane lipid metabolism, as well as some extracellular nucleotides, including uridine triphosphate (UTP) and adenosine triphosphate (ATP) [15–17], play an important role as homing factors for HSPCs, in addition to SDF-1.

Besides these novel, still underappreciated, homing factors, several mediators are upregulated in BM conditioned for transplantation that may positively enhance (prime) the responsiveness of CXCR4^+^ HSPCs to an SDF-1 gradient [18, 19]. This is biologically significant because, as mentioned previously, myeloablative conditioning for transplantation induces a highly proteolytic microenvironment in BM that leads to proteolytic degradation of SDF-1 and attenuates its chemotactic gradient [9]. Therefore, all these SDF-1-CXCR4 axis-sensitizing factors counteract a decrease in the active SDF-1 gradient. These important priming factors or modulators of the SDF-1-CXCR4 axis include elements of innate immunity, such as cleavage fragments of the third component (C3) of the complement cascade (CC), antimicrobial cationic peptides, such as cathelicidin (LL-37) and *β*2-defensin, and the eicosanoid prostaglandin E2 (PGE2) [18–23]. While C3 cleavage fragments (short-lived C3a and its long-lived derivative _desArg_C3a), LL-37, and *β*2-defensin increase the chemotactic responsiveness of HSPCs to very shallow SDF-1 gradients by promoting incorporation of the CXCR4 receptor into membrane lipid rafts, which are necessary for optimal activation of this receptor [18, 19], PGE2 upregulates the expression level of this receptor on HSPCs [22, 24]. In both of these situations, the CXCR4^+^ HSPCs are able to respond more robustly to SDF-1.

**Figure 1:**
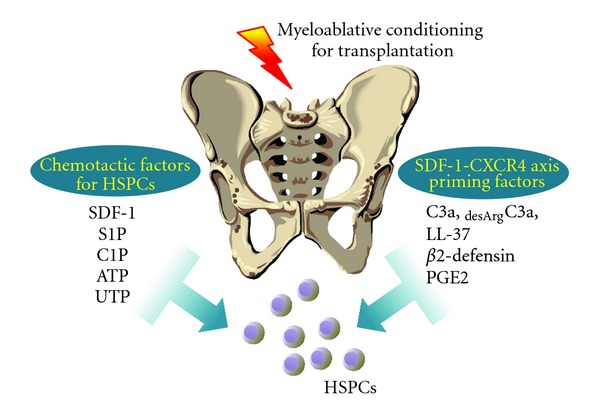
Homing of HSPCs to BM—the involvement of new chemotactic and priming factors. Evidence has accumulated that HSPCs home to BM in response not only to SDF-1 but also in response to some bioactive lipids (S1P and C1P), as well as extracellular nucleotides (ATP and UTP). The responsiveness of HSPCs to an SDF-1 gradient is also positively modulated by several priming molecules, including peptides of the cationic antimicrobial peptide (CAMPs) family, and PGE2.

The mechanisms that govern homing of HSPCs to BM have been the subject of several recent reviews [3, 23–27]. However, here we will focus on novel factors that play a role in SDF-1-independent BM homing as well as factors that modulate the activity of the SDF-1-CXCR4 axis and are involved in homing of HSPCs. What is most important, all of these factors become upregulated in BM conditioned for transplantation. Thus, the existence of these factors provides a novel and complex picture of the homing process that will be presented and discussed in more detail herein.  Figure 1 shows a novel view of HSPC homing in response to SDF-1 and all other chemoattractants involved in this process as well as the involvement of the SDF-1-CXCR4 axis priming factors.

## 2. The Role of SDF-1 in Developmental Migration of HSPCs and in Adult Hematopoiesis

HSPCs migrate during embryonal development, colonizing different organs where hematopoiesis is initiated. First, definitive HSPCs are identified in the so-called aorta-gonado-mesonephros (AGM) region, and in the second trimester of gestation they colonize fetal liver, which is a major hematopoietic organ at this stage of development [28–32]. Subsequently, at the beginning of the third trimester of gestation, HSPCs leave the fetal liver and colonize the developing BM, which will become a major hematopoietic organ for the rest of mammalian life [33, 34].

The role of SDF-1 in developmental colonization of the BM microenvironment was convincingly demonstrated in SDF-1 and CXCR4 knockout animals [6, 35, 36]. These studies revealed that murine embryos that lack SDF-1 or CXCR4 display defects in BM development [36], as well as other defects in heart, brain, and large-vessel development that contribute to their lethal phenotype [37–40], and die *in utero*. However, except for a defect in B-lymphocyte lineage development, they have normal fetal liver hematopoiesis [36–40].

These studies on SDF-1^−/−^ and CXCR4^−/−^ embryos revealed two important points. First, colonization of fetal liver during embryogenesis by AGM-derived HSPCs is not governed by the SDF-1-CXCR4 axis, and second, SDF-1 is required for proper migration of HSPCs from fetal liver to BM. The fact that murine embryos with CXCR4- or SDF-1-deficiency have a normal number of myeloid HSPCs in fetal liver [6, 37–43], which is colonized by HSPCs migrating from the AGM region, suggests that this process is mediated by other chemoattractants. Taking into consideration the important role of S1P in the development of several tissues during embryogenesis [44, 45], it is likely that S1P compensates for the SDF-1-CXCR4 deficiency in these animals during developmental migration of HSPCs. This, however, needs further study. It is also obvious that at some point in development, HSPCs increase their dependence on SDF-1-mediated chemotaxis.

Interestingly, as mentioned previously, fetal liver-derived CXCR4^−/−^ cells may still engraft in lethally irradiated mice and radioprotect these animals [1, 42, 46]. However, it has been demonstrated that irradiation chimeras created with CXCR4^−/−^ HSPCs display some defects in expansion and retention of HSPCs in BM [42]. This fact pinpoints the requirement of the SDF-1-CXCR4 axis not only in retention of HSPCs in BM but also in proper adult hematopoiesis and maintenance of a quiescent HSC pool [42, 46]. In this process, the SDF-1-CXCR4 axis plays a crucial role, together with other factors, such as Very Late Antigen-4 (VLA-4, also known as *α*_1_ *β*_4 _ integrin), expressed on HSPCs, and Vascular Adhesion Molecule 1 (VCAM-1, also known as CD106) expressed in the BM niche [47–49].

SDF-1 has been reported as being expressed in both osteoblastic and endothelial stem cell niches by osteoblasts and endothelial cells, respectively [25, 50]. In support of this finding, morphological studies revealed that HSCs are found in the BM microenvironment in contact with the cells expressing high amounts of SDF-1, which are called CXCL12-(another name for SDF-1) abundant reticular (CAR) cells. In particular, CAR cells surround sinusoidal endothelial cells and are located near the endosteum as part of the endothelial and osteoblastic niches, respectively [50, 51]. SDF-1 is also secreted by BM stromal cells, including nestin^+^ cells [51, 52].

Expression of SDF-1 is regulated at the transcriptional level by hypoxia-inducible factor 1 alpha (HIF-1*α*), which is upregulated in BM conditioned for transplantation in response to myeloablative treatment [53]. Nevertheless, despite upregulation of SDF-1 expression at the mRNA level, the chemotactic gradient of SDF-1 protein in BM may be attenuated by several proteolytic enzymes, whose expression is induced in BM after myeloablative conditioning for transplantation [9]. To ameliorate this effect, several mechanisms have been identified that compensate for a decrease in the SDF-1 gradient, which will be discussed in the following section.

## 3. Priming Factors That Responsiveness of CXCR4^+^ HSPCs to an SDF-1 Gradient

As mentioned previously, the biological activity of SDF-1 decreases in BM due to the induction of a proteolytic microenvironment after conditioning for transplantation, as seen, for example, after lethal irradiation [9]. In an elegant study, it has been shown that a few amino acids located at the N-terminus of SDF-1 that are crucial for the biological activity of this peptide may be removed by metalloproteinase-2 (MMP-2) or MMP-9 [54]. This proteolytic processing of SDF-1 completely inhibits its chemotactic properties [9]. However, as shown in Figure 1, at the same time several factors have been reported to enhance or sensitize the responsiveness of HSPCs to an SDF-1 gradient. These factors include elements of innate immunity, such as cleavage fragments of C3 [18], cationic antimicrobial peptides, such as cathelicidin (LL-37) and *β*2-defensin [19–21], and prostaglandin E2 (PGE2) [22–24], a member of the eicosanoid family.

**Figure 2:**
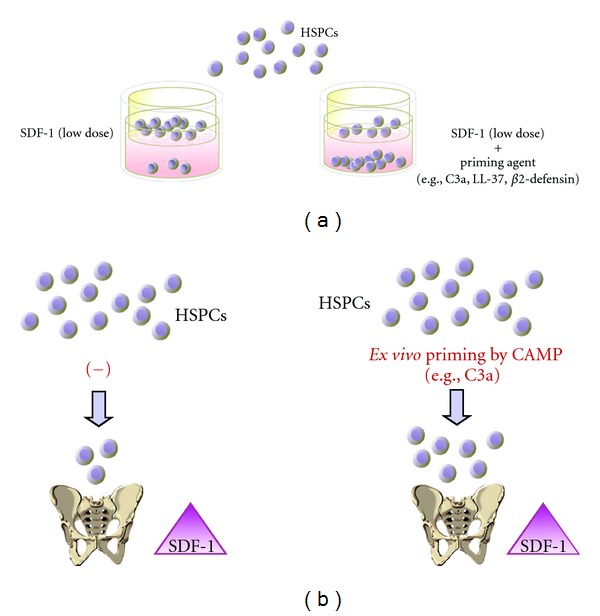
A priming effect increases the responsiveness of HSPCs to shallow SDF-1 gradients. The overall scheme of chemotactic assays performed in the transwell system to evaluate the HSPC priming phenomenon (a). In the presence of a priming agent (e.g., cationic antimicrobial peptides (CAMPs), such as C3a or cathelicidin (LL-37) or *β*2-defensin), HSPCs respond more robustly to low doses of SDF-1 [20, 55]. This phenomenon is currently being tested in the clinic, where UCBs are exposed *ex vivo* to a priming agent (C3a) before transplantation (b).

**Figure 3:**
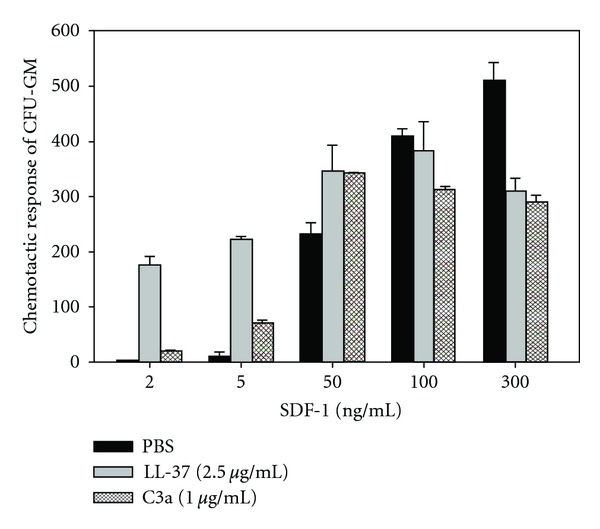
Cationic antimicrobial peptides (CAMPs) C3a and LL-37 enhance the responsiveness of murine BM- and human UCB-derived HSPCs to an SDF-1 gradient: Chemotaxis of murine BM CFU-GM in response to different concentrations of SDF-1 with and without C3a or LL-37 [20, 55]. Values are the fold increase in the number of migrated cells compared to the number of migrated cells in medium alone. Gray bars indicate the presence of LL-37 (2.5 *μ*g/mL), cross-hatched bars the presence of C3a (1 *μ*g/mL) in the lower transwell chambers, and black bars indicate PBS only. The data represent the combined results from three independent experiments performed in duplicate per group (*n* = 6).

This priming effect can easily be evaluated *in vitro* in the transwell migration assay, where two chambers (an upper chamber containing the tested cells and a lower chamber containing chemoattractant) are separated by a porous membrane that allows transmigration of cells that respond to the chemotactic gradient (Figure 2). Cells that respond to this gradient migrate and subsequently accumulate in the lower chamber. Figures 2 and 3 demonstrate that the chemotaxis of HSPCs to a shallow SDF-1 gradient may be significantly enhanced in the presence of cationic antimicrobial peptides (CAMPs) [20, 55]. The role of these priming factors in modulating the SDF-1-CXCR4 axis will be discussed hereinafter.

### 3.1. C3 Cleavage Fragments

It has been demonstrated that the CC, as an evolutionarily old danger-sensing mechanism, becomes activated during conditioning for transplantation by radio- and chemotherapy [9]. The third component of the CC (C3) is an abundant protein in PB plasma (1 mg/mL) and becomes cleaved during CC activation by both classical and alternative pathways [56]. The C3 cleavage leads to release of liquid-phase cleavage fragments, the C3a and _des-Arg_C3a anaphylatoxins [57]. Liquid-phase anaphylatoxin C3a has a short half-life in plasma and is processed by serum carboxypeptidase N to _des-Arg_C3a, which is a long-half-life cleavage product.

Previous work on C3^−/−^ mice revealed that these animals are hematologically normal under steady-state conditions and display a significant delay in hematopoietic recovery from either irradiation or transplantation of wild type (WT) HSPCs [55, 58, 59]. Specifically, transplantation of histocompatible wild type (WT) Sca-1^+^ cells into C3^−/−^ mice resulted in (i) a decrease in day 12 colony forming units in spleen (CFU-S), (ii) a 5–7-day delay in platelet and leukocyte recovery, and (iii) a reduced number of BM hematopoietic clonogenic progenitors at day 16 after transplantation. The fact that HSPCs from C3^−/−^ mice engrafted normally into irradiated WT mice suggests that there was a defect in the hematopoietic environment of C3^−/−^ mice and not some intrinsic defect of C3^−/−^ mouse-derived HSPCs [18, 58].

Since C3^−/−^ mice cannot activate/cleave C3, the C3 fragments C3a and _des-Arg_C3a were examined for a role in HSPC engraftment, and we found that C3a and _des-Arg_C3a increase CXCR4 incorporation into membrane lipid rafts, thus potentiating HSPC responsiveness to SDF-1 gradients [59, 60].

Lipid rafts are membrane domains rich in sphingolipids and cholesterol, which form a lateral assembly in a saturated glycerophospholipid environment. The raft domains are known to serve as moving platforms on the cell surface and are more ordered and resistant to nonionic detergents than other areas of the membrane [61]. These domains are also good sites for crosstalk between various cellular signaling proteins. For example, it has recently been reported that small guanine nucleotide triphosphatases (GTPases), such as Rac-1 and Rac-2, which are crucial for engraftment of hematopoietic cells after transplantation, are associated with lipid rafts on migrating HSPCs [62–64]. Therefore, since the CXCR4 receptor is a lipid raft-associated protein, its signaling ability is enhanced if it is incorporated into membrane lipid rafts, where it can better interact with several signaling molecules, including the small GTPase Rac-1. This colocalization of CXCR4 and Rac-1 in lipid rafts facilitates GTP binding and activation of Rac-1 [62, 65–67]. Thus, the generation of C3 cleavage fragments in the BM microenvironment may somehow act as a mechanism that increases the responsiveness of HSPCs to an SDF-1 gradient when it is degraded by a proteolytic microenvironment [18]. In C3-deficient mice this phenomenon is attenuated, explaining why these animals show delayed engraftment. In this context, increases in C3a or _desArg_C3a levels in BM after myeloablative conditioning [18] can be envisioned as one of the mechanisms that promote homing of HSPCs (Figures 1–3).

### 3.2. Cationic Antimicrobial Peptides (CAMPs)

CAMPs are host-defense peptides and are an evolutionarily conserved component of the innate immune response [68–71]. CAMPs have been demonstrated to kill bacteria, enveloped viruses, fungi, and even transformed or cancerous cells but affect only the organization and not the viability of the eukaryotic cell membrane. The selective effects of CAMPs (e.g., eukaryotic membrane perturbation and prokaryotic killing) are known to be dependent on characteristics of cell membranes [21, 68–71]. Prokaryote cell membranes are susceptible to strong electrostatic and hydrophobic interactions with these natural antibiotics. In contrast, cell membranes of eukaryotic cells, because of high cholesterol content and weak hydrophobic interactions with cationic peptides, are more resistant to the potentially toxic effects of these peptides. One of the properties of CAMPs that we identified is their ability to enhance or prime the responsiveness of cells to an SDF-1 gradient. Interestingly, the C3a and _desArg_C3a anaphylatoxins mentioned previously share several properties with CAMPs [72].

Cathelicidin (LL-37) and *β*2-defensin belong to the CAMP family and like C3a, as mentioned before, increase (positively prime) the responsiveness of HSPCs to an SDF-1 homing gradient (Figure 1). The molecular explanation of this phenomenon is the same as in the case of C3a: CAMPs promote the incorporation of CXCR4 into membrane lipid rafts [59, 60]. Since, as mentioned before, membrane lipid rafts assemble together several signaling molecules, incorporation of CXCR4 into lipid rafts facilitates signaling, and thus CXCR4 is activated more efficiently in the presence of low doses of SDF-1.

Further studies are needed to see whether, in addition to CXCR4, receptors for other chemoattractants of HSPCs that we will discuss hereinafter, such as S1P, C1P, ATP, and UTP, are also lipid raft-regulated and whether CAMPs enhance their incorporation into membrane lipid rafts. Of note, it has been reported that stimulation of S1P receptor type 1 (S1P_1_) by its agonist, FY720, increases the responsiveness of HSPCs to an SDF-1 gradient [73]. However, this phenomenon probably occurs because of intercellular crosstalk between CXCR4 and S1P_1_. Since a receptor for another bioactive lipid, C1P, has not yet been identified, it is not clear whether C1P signaling is also lipid raft-regulated. However, data indicate that this receptor is expressed on HSPCs and is sensitive to pertussis toxin, which suggests that, like S1P, it is a G_*α*i_ protein-coupled receptor [9]. Also, purinergic receptors for ATP and UTP are G_*α*i_ protein-coupled receptors, and the possibility of modulation of the activity of these receptors by C3a, LL-37, or *β*2-defesin requires further study.

### 3.3. Prostaglandin E2

 In addition to cationic peptides, prostaglandin E2 (PGE2) has also been purported to increase the responsiveness of HSPCs to an SDF-1 gradient [22, 23]. The mechanism of PGE2 influence on this process, however, is not lipid raft dependent. As previously reported, PGE2 plays an important role in homing of HSPCs by upregulating the expression of CXCR4 on the surface of HSPCs, and this seems to be the most likely mechanism responsible for increasing chemotaxis in response to an SDF-1 gradient after pretreatment of HSPCs by PGE2 [22–24, 74]. In further support of a role for PGE2 in homing, it has recently been reported that the level of this eicosanoid is significantly upregulated in BM conditioned for hematopoietic transplantation by lethal irradiation [9].

In addition to the CAMPs and PGE2, hyaluronic acid [75] and thrombin [76] have also been reported to increase the responsiveness of HSPCs to an SDF-1 gradient. The exact mechanism of this priming effect, however, has still not been elucidated but is most likely mediated by the interaction of hyaluronic acid with integrin receptors on HSPCs that are present in lipid rafts. This possibility, however, needs further study.

## 4. The Bioactive Sphingolipids S1P and C1P as Novel BM Homing Factors

Sphingophospholipids are important components of cell membranes and are derived from the aliphatic amino alcohol sphingosine and its acylated derivative ceramide [27, 77–79]. Both sphingosine and ceramide are precursors for the bioactive derivatives sphingosine-1-phosphate (S1P) and ceramide-1-phosphate (C1P), which strongly chemoattract HSPCs [9].

S1P is a product of two sphingosine kinases (SK1 and SK2), is released from cells by a transporter-facilitated process, and interacts with at least five G_*α*i_, G_12/13_, and G_q_ protein-coupled seven-transmembrane-spanning receptors, S1P_1–5_, on the surface of target cells [80, 81]. While S1P_1_ and S1P_3_ receptors are most important in promoting the migration of HSPCs, S1P_2_ may have an opposing function [82–84]. Of note, S1P_1–5_ receptors are rapidly internalized from the cell surface after binding S1P, which is similar to the internalization of CXCR4 after binding SDF-1. Accordingly, S1P has been identified as a chemottractant for hematopoietic progenitor cells [11, 12, 56], a regulator of trafficking of T lymphocytes between lymphoid organs and PB [85–87], a factor involved in egress of early B-lymphoid cell progenitors from BM [88, 89], and a regulator in the trafficking of myeloid progenitors between BM and peripheral organs [89]. We and others have recently demonstrated that S1P plays a pivotal role in pharmacological mobilization and egress of HSPCs from BM into peripheral blood (PB) [12, 90] and since it is unregulated in BM conditioned for transplantation may also probably chemoattract HSPCs to BM [9].

Another bioactive sphingolipid, C1P, is structurally related to S1P and can be generated by phosphorylation of ceramide (N-acyl sphingosine) by ceramide kinase (CERK) [91]. Unlike ceramide (which is often proapoptotic), C1P has been reported to promote cell growth, survival, and migration through an unknown receptor-initiated signaling pathway that is pertussis toxin-sensitive and therefore likely to involve a G_*α*i_ protein-coupled seven-transmembrane-spanning receptor [14, 92]. The receptors for C1P, however, have not yet been identified, though they are clearly distinct from the known S1P receptors. C1P was initially identified as a chemottractant for monocytes [13] and, as has recently been demonstrated, is also an important, novel, and potent chemotactic factor involved in the homing of HSPCs to BM [9].

There are some obvious differences in biological availability between these bioactive lipids. While S1P is released from cells as an important signaling molecule and in PB is transported by erythrocytes, platelets, albumin, and high density lipoproteins (HDL), C1P is an intracellular second messenger released from leaky damaged cells and is also abundant in plasma in the HDL fraction [14]. While considering chemotactic gradients of S1P and C1P, one has to remember that both bioactive lipids must be present in biological fluids as free, unbound molecules in order to have a chemotactic potential [9].

Importantly, both S1P and C1P are upregulated in the BM microenvironment after myeloablative conditioning of BM for transplantation [9]. For example, our recent mass spectrometry (MS) analysis revealed that the major isoforms of C1P and S1P were detected at higher concentrations in supernatants harvested from irradiated BM. Taken together with their potent chemotactic effects, these changes in concentration of bioactive lipids in BM after myeloablative conditioning for transplantation suggest that these factors play an important role in the homing process for HSPCs [9].

However, both bioactive lipids have a limited half-life, with S1P degraded by several enzymes, such as S1P lyase (SPL), lipid phosphate phosphatases (LPP1–3), and S1P-specific phosphatases (SPP1 and SPP2), while C1P is degraded by LPP1–3 [93–101]. These pathways may terminate the effects of S1P and C1P on HSPC migration. Therefore, further studies are needed to evaluate the changes in kinetics of the response to S1P and C1P gradients in BM after myeloablative treatment. 

**Figure 4:**
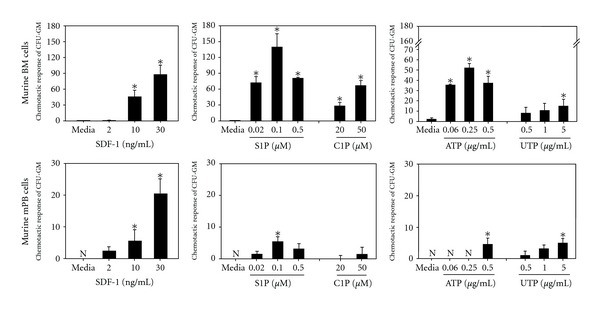
Responsiveness of murine bone marrow-(BM-) and mobilized peripheral blood-(mPB-) derived HSPCs to SDF-1, S1P, C1P, ATP, and UTP gradients. The difference in responsiveness of HSPCs isolated from BM versus mPB could be explained by a fact that HSPCs in mPB are exposed to high concentration of bioactive lipids and extracellular nucleotides, which may lead to downregulation or desensitization of appropriate receptors. After internalizing these receptors, HSPCs need some time to reexpress functional receptors on the cell surface to recover their responsiveness to these chemoattractants (data not published, *P* < 0.01).

Furthermore, as mentioned previously, S1P_1–5_ receptors and, most likely, still-unknown C1P receptors are downregulated on the surface of HSPCs in the presence of S1P and C1P, respectively [27]. This may explain why HSPCs harvested from mPB previously exposed to high S1P plasma concentrations do not respond robustly to S1P and C1P gradients compared to BM-derived HSPCs (Figure 4). After internalizing S1P receptors, HSPCs need some time to reexpress functional receptors on the cell surface to recover their responsiveness [27].

Finally, in addition to S1P and C1P, we tested other bioactive lipids. We observed that, in contrast to S1P and C1P, other members of the bioactive lipid family, such as lysophosphatidic acid (LPA) and lysophosphatidylcholine (LPC), do not show chemotactic activity against HSPCs. Thus, S1P and C1P seem to play a unique role among evaluated so far members of the family of bioactive lipids. 

## 5. Purinergic Nucleotides as Underappreciated Homing Factors

Evidence has accumulated that extracellular-secreted 5′-nucleotide triphosphates (ATP and UTP) are important biological mediators involved in cell proliferation, survival, and cell trafficking [102, 103]. In one elegant work, it was shown that ATP and UTP stimulate, in synergy with some cytokines, expansion of HSCs that repopulate BM after transplantation in an immunodeficient mouse model [16]. These nucleotides have also been described as chemotactic factors in several types of cells, including granulocytes and monocytes [15–17, 104]. Significantly, as recently demonstrated for UTP and to a lesser extent ATP, they modulate trafficking of HSCs and their homing to BM niches [16]. Thus, extracellular nucleotides may provide a powerful tool to modulate the function of HSPCs. Interestingly, they display the opposite effects on human acute myeloblastic leukemia cells to that which is observed for normal HSCs [105], by inhibiting proliferation, migration, and engraftment of these cells in immunodeficient mice.

Both UTP and ATP activate target cells by activating P2X and P2Y purinergic receptors and P2Y seven-transmembrane-spanning G_*α*i_-coupled receptors are most important in mediating the responses of these novel BM homing factors [17]. Since both ATP and UTP are released from damaged cells, as seen after myeloablative conditioning for transplantation, this explain why they play an important role together with other factors in the homing of transplanted HSPCs (Figure 1).

## 6. Future Directions

Evidence has accumulated that the chemotactic responsiveness of HSPCs to several homing gradients could be modulated by *ex vivo* manipulations. One potential strategy is taking advantage of the HSPC-priming approach. For instance, the possibility of accelerating or enhancing the homing of HSPCs by *ex vivo* exposure of cells in the graft to C3a before infusion into the patient is currently being evaluated in an ongoing clinical trial (Masonic Cancer Center, University of Minnesota). Another interesting molecule that should be tested in the clinical setting as a potential priming factor is the cathelicidin LL-37. The advantage of LL-37 is that it is a physiological factor secreted by BM stromal cells, and, as shown in Figure 3, is a more potent priming factor than C3a [20]. Another possible *ex vivo* manipulation of HSPCs in grafting is exposure to PGE2 in order to upregulate expression of CXCR4 to enhance the homing of transplanted cells [24]. This strategy is also currently being evaluated in clinical trial. Overall priming strategies would be important in these clinical situations when a number of HSPCs to be transplanted are limited as seen, for example, in UCB transplants.

Furthermore, since HSPCs in the plasma of mobilized PB and umbilical cord blood are exposed to relatively high S1P and C1P levels (~1 *μ*M) and the S1P receptors and most likely C1P ones become internalized mPB-derived HSPCs, in contrast to BM-derived HSPCs, they respond weakly to bioactive lipids gradients (Figure 4). To reestablish expression of these receptors on the surface of HSPCs and their responsiveness to S1P and C1P gradients, HSPCs should be exposed to culture medium free of both bioactive lipids before transplantation. Furthermore, since several agonists and inhibitors of S1P receptors promoting (S1P_1_ and S1P_3_) and inhibiting (S1P_2_) homing and enzymes involved in synthesis or degradation of S1P and C1P are available [44, 82–84], this opens up new possibilities for positively modulating the responsiveness of HSPCs to BM S1P and C1P gradients. These tools may lead to more efficient strategies to improve homing of HSPCs, and these approaches are currently being tested in murine models.

Since mPB-derived HSPCs show similar relatively weak responsiveness to ATP and UTP gradients (Figure 4), in order to reestablish their responsiveness to gradients of these extracellular nucleotides, HSPCs should be also probably exposed to culture medium free of these factors before transplantation.

In addition to aforementioned strategies there are also other possibilities to manipulate HSPCs to enhance their homing that were out of scope of this paper. Accordingly, based on observations that the SDF-1 interaction with CXCR4^+^ HSPCs is attenuated by the dipeptidyl-peptidase CD26, inhibition of CD26 on HSPCs could enhance the chemotactic responsiveness to an SDF-1 gradient [7]. Another interesting strategy is modification of adhesion molecules on HSPCs by *ex vivo* treatment with fucosyltransferase that increases level of fucosylation of these receptors [106]. As it has been demonstrated human HSPCs after blockage of CD26 or after fucosylation of adhesion molecules home and subsequently engraft better on immunodeficient mice [104].

These developments suggest that hematological transplantology can take advantage of basic research into novel chemoattractants and priming mechanisms that facilitate homing of HSPCs and translate these observations into more efficient clinical protocols.
